# Transcription Termination Defective Mutants of Rho: Role of Different Functions of Rho in Releasing RNA from the Elongation Complex

**DOI:** 10.1016/j.jmb.2007.06.013

**Published:** 2007-08-24

**Authors:** Jisha Chalissery, Sharmistha Banerjee, Irfan Bandey, Ranjan Sen

**Affiliations:** Laboratory of Transcription Biology, Centre for DNA Fingerprinting and Diagnostics, ECIL Road, Nacharam, Hyderabad-500076, India

**Keywords:** RNAP, RNA polymerase, EC, elongation complex, %RT, read-through efficiency, RNA polymerase, Rho, NusG, transcription termination, mutagenesis

## Abstract

The transcription termination factor Rho of *Escherichia coli* is a RNA binding protein which can translocate along the RNA and unwind the RNA:DNA hybrid using the RNA-dependent ATPase activity. In order to investigate the involvement of each of these functions in releasing RNA from the elongation complex, we have isolated different termination defective mutants of Rho by random mutagenesis, characterized them for their different functions and established the structure–function correlations from the available structural data of Rho. These mutations are located within the two domains; the N-terminal RNA binding domain (G51V, G53V, and Y80C) and in the C-terminal ATP binding domain (Y274D, P279S, P279L, G324D, N340S, I382N) including the two important structural elements, the Q-loop (P279S, P279L) and R-loop (G324D). Termination defects of the mutants in primary RNA binding domain and Q-loop could not be restored under any conditions that we tested and these were also defective for most of the other functions of Rho. The termination defects of the mutants (Y274D, G324D and N340S), which were mainly defective for secondary RNA binding and likely defective for translocase activity, could be restored under relaxed *in vitro* conditions. We also show that a mutation in a primary RNA binding domain (Y80C) can cause a defect in ATP binding and induce distinct conformational changes in the distal C-terminal domain, and these allosteric effects are not predictable from the crystal structure. We conclude that the interactions in the primary RNA binding domain and in the Q-loop are mandatory for RNA release to occur and propose that the interactions in the primary RNA binding modulate most of the other functions of Rho allosterically. The rate of ATP hydrolysis regulates the processivity of translocation along the RNA and is directly correlated with the efficiency of RNA release. NusG improves the speed of RNA release and is not involved in any other step.

## Introduction

Once the process of transcription is initiated, RNA polymerase (RNAP) makes a stable elongation complex with the DNA and the nascent RNA.^1–3^ Elongation complex (EC) only dissociates in response to *cis* or *trans* termination signals. Distinguished by their mechanism and structural features, there are two types of terminators in the *Escherichia coli* genome. (a) Intrinsic terminators characterized by a GC-rich inverted repeat followed by a oligo(dT) stretch that induce RNA polymerase to disengage RNA; and (b) the factor-dependent terminators, which depend on an essential protein factor, called Rho, for termination.^4,5^

Rho is a homo-hexameric RNA/DNA helicase or translocase that dissociates RNA polymerase from DNA template and releases RNA. RNA-dependent ATPase activity of Rho provides free energy for these activities.^6–8^ The primary RNA binding site (residues 22–116) can bind to a single-stranded DNA molecule as well as a single-stranded RNA molecule and is responsible for recognizing the Rho utilization (*rut*) site.^9^ Amino acid residues 179–183 form the P-loop, a highly conserved region among the RecA family of ATPases and is involved in ATP binding and ATPase activity.^10^ The Q-loop and R-loop have been defined as the secondary RNA binding sites. The Q-loop is formed by an eight-residue segment within 278–290 amino acid residues,^10–13^ while the R-loop spans between 322–326 amino acid residues.^11,14,15^

Rho-dependent termination involves a series of sequential events. At first Rho binds to the C-rich regions of the nascent RNA, called the *rut* site^16–18^ through its primary RNA binding domain. This binding leads to the positioning of the RNA into the secondary RNA binding domain, which in turn activates the ATPase activity. It utilizes the free energy derived from the ATP hydrolysis for its translocase/RNA–DNA helicase functions,^6,7^ which eventually leads to the release of the RNA from the elongation complex. It is commonly believed that the translocase/helicase activities of Rho is instrumental in pulling out RNA from the elongating RNA polymerase.^7^ However, it is not well understood how each of these events is related to the RNA release process.

Here, we investigated the involvement of different functions of Rho in releasing the RNA from the elongation complex. We randomly mutagenized the *rho* gene, isolated the termination defective mutants, characterized each of them for their defects in other functions and established a structure–function relationship of the effects of these mutations based on the recent closed ring structure of Rho.^19^ This is in contrast to earlier mutagenesis approaches which were only restricted to identify the different functional domains of Rho.^13,14,20^ We also established an *in vitro* experimental set-up to measure the Rho-mediated RNA release from a stalled EC, uncoupled from the transcription elongation process. This set-up enabled us to find the conditions under which some of these mutants regained the efficiency of RNA release and to understand the specific roles of ATP and NusG in the process of Rho-dependent termination. We concluded that the mutations in the N-terminal primary RNA binding domain can exert allosteric effects in the distal C-terminal domain, and this domain may act as a “master switch” that governs all the other functions of Rho. The primary RNA binding domain and the Q-loop are the most crucial structural elements for the RNA release function. Altered interactions in the secondary RNA binding domain only reduce the rate of ATPase activity which most likely slows down the translocation rate of Rho along the RNA. The rate of ATP hydrolysis is directly correlated with the efficiency of RNA release and involvement of NusG is restricted only to the final step of RNA release from the EC.

## Results

### Isolation of termination defective mutants of Rho

We have randomly mutagenized the whole *rho* gene and screened for mutants defective for termination at two Rho-dependent terminators (see Materials and Methods). After screening about 100,000 colonies, we isolated nine unique mutations in Rho which were severely defective for termination. These mutations are G51V, G53V, Y80C, Y274D, P279S, P279L, G324D, N340S and I382N. G51V and I382N mutations were independently isolated at least six times. G51V and G53V were also isolated earlier from a localized mutagenesis screen of the primary RNA binding domain.^20^ Although the positions of the mutations indicate that the mutagenesis was random, the classical polarity suppressor mutations^21^ were not isolated by this method, the reason of which is not clear to us. Termination defects exhibited by these mutations on three Rho-dependent terminators (defect for *nutR/TR*_*1*_ terminator is described below) suggest that the defect is not specific for a particular terminator. We also observed that mutants other than G51V, Y80C and Y274D caused a growth defect at 42 °C.

### *In vivo* termination defects of the Rho mutants

To get a quantitative measure of the termination defects of the Rho mutants, the ratio of β-galactosidase made from a *lacZ* reporter fused to a Rho-dependent terminator (P_*lac*_-H-19B *nutR*/*TR*_*1*_-*lacZYA*) to that without the terminator (P_*lac*_-*lacZYA*) was measured (read-through efficiency=%RT). This Rho-dependent terminator is derived from the *nutR-cro* region of a lambdoid phage H-19B^22^ and found to behave similar to the *nutR/T*_*R1*_ of λ phage. These reporter fusions were present as a single copy prophage (see Materials and Methods and Table 6 for the strains). Different strains with these reporter fusions were transformed with the plasmids carrying the Rho mutants. The measurements were done in the presence of either wild-type (WT) or B8 (rpoB, Q513P) RNA polymerase in the chromosome and either in the presence of a WT or chromosomal deletion of Rho.

Compared to WT Rho, all the mutants showed a significant defect in termination as evident from the 10 to 20-fold increase in terminator read-through efficiency (Table 1, column 4). Even in the presence of a WT copy of Rho, the read-through efficiency of the mutants was significantly high (Table 1, column 7). This could be attributed to the multi-copy (copy number of pCL1920 is ∼ 5)^23^ dominance of the mutant Rho over WT Rho. The partial suppression of the defects could also arise from the mixing of WT and mutant protomers of Rho.

The kinetic coupling model of Rho-dependent termination predicts that a slow moving RNA polymerase can suppress the effect of defective Rho.^24^ So we wanted to know whether the defects of these mutants could be suppressed by B8 (rpoB, Q513P), a slow elongating RNA polymerase.^25^ Similar measurements of termination read-through in the presence of B8 RNAP showed that defects of none of the Rho mutants were suppressed by the “slow” RNAP (Table 1, column 10), which is in contrast to earlier observations of different Rho mutants being suppressed by this RNAP.^24,26^ According to the kinetic coupling model, slow RNAP will suppress only the Rho mutants which are defective in translocation along the RNA. Thus, either these mutants translocate too slow to catch up B8 RNAP or these mutants are defective in other steps of Rho-dependent termination. It is also noted that none of these mutants were compatible with a fast moving RNAP, B2 (rpoB, H526Y). It was difficult to obtain transformants in the strain having B2 RNAP in the chromosome with all the plasmids bearing Rho mutants. The defects of these Rho mutants might have amplified in the presence of a fast moving RNAP that caused this lethality. We also observed increased read-through activity with the slow RNAP (B8), which was unexpected (Table 1, column 10) and the reason for this is not clear.

### *In vitro* termination defects of the Rho mutants

**Figure 1:**
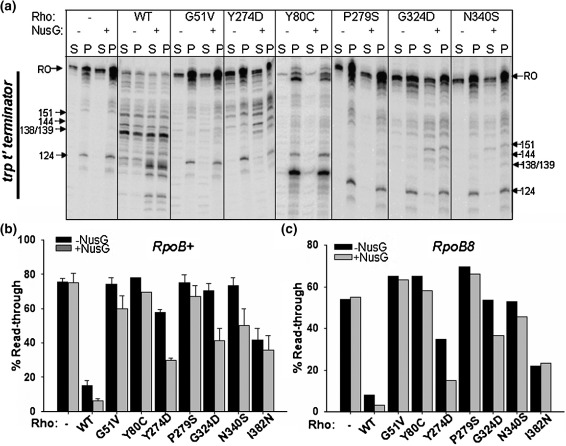
*In vitro* termination defects of the mutant Rho proteins. (a) Autoradiogram of the *in vitro* transcription termination by different Rho mutants both in the absence and presence of 200 nM NusG. Transcription was initiated from T7A1 promoter. The DNA template was immobilized on magnetic beads. Rho-dependent RNA release starts from 100 nt downstream of the transcription start site in the *trp t'* terminator region (as indicated). Major termination positions are marked. Half of the supernatant is denoted as S (released RNA) and the rest of the reaction mixture containing both supernatant and pellet is denoted as P (total RNA). RO denotes the run-off product. The read-through efficiency (RT) for each case was calculated from the P lanes as: RT=[RO product]/[RO product + all the products ≥ 100 nt]. (b) Measured RT for each of the mutants is shown as bar diagrams using WT RNA polymerase. Higher read through efficiencies of the Rho mutants reflect their termination defect. (c) Read through efficiencies measured in the presence of B8 RNA polymerase.

*In vitro* transcription termination activity of the Rho mutants was studied at the *trp t'*terminator^21,27,28^ using purified WT and all the mutant Rho proteins, except G53V and P279L. We could not purify G53V and P279L mutants because they formed inclusion bodies under overexpression conditions. A linear DNA template with the *trp t'* terminator cloned downstream to the strong T7A1 promoter was used for transcription assays (see Materials and Methods). In order to observe the released RNA at the Rho-dependent termination points, the templates were immobilized on streptavidin-coated magnetic beads. In the assays, the supernatant fractions contain the released RNA. The WT Rho protein showed about 85% efficiency in termination. Consistent with their *in vivo* phenotype, all the Rho mutants except I382N showed significantly reduced termination efficiency (Figure 1(a) and (b)). The presence of NusG during the chase improved the termination efficiency of Y274D, G324D and N340S by about twofold (Figure 1(a) and (b)). NusG, however, did not bring about early termination in these mutants as observed for the WT Rho (Figure 1(a)).

In order to validate the *in vivo* observation that the termination defects of the Rho mutants could not be suppressed by B8 RNAP, *in vitro* transcriptions were also carried out with the B8 enzyme. Consistent with the *in vivo* data (Table 1), the *in vitro* assays (Figure 1(c)) also showed that B8 RNAP does not improve the termination efficiency of the mutants significantly, except for Y274D. For Y274D and G324D mutants, termination efficiency was improved ∼ 1.5-to twofold in the presence of NusG. These mutants (and N340S; see Figure 1(a) and (b)) might have defects in the RNA release step(s) from the EC, and NusG is likely to be involved in this step during the Rho-dependent termination process.

### Location of the mutations on the crystal structure of Rho and prediction of functional defects

**Figure 2:**
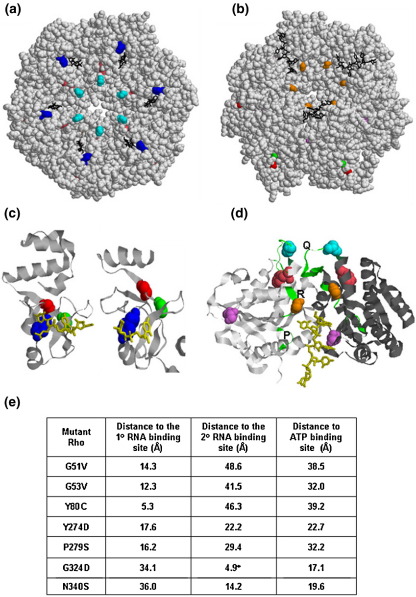
Location of the mutations on the crystal structure of Rho. In (a) and (b) the space-filled model of hexameric closed ring structure of Rho was generated from the available co-ordinates of the dimeric unit of Rho (2HT1^19^) and the Figures were prepared using RASMOL. Positions of the mutations are indicated. The color codes are as follows. G51V, red; G53V, green; Y80C, blue; Y274D, pink; P279S/P279L, cyan; G324D, orange; N340S, violet. The position of I382N is not resolved in this structure. In (a) the view is from primary RNA binding domain and that in (b) is from secondary RNA binding domain. (c) The primary RNA binding domain of Rho generated from the co-ordinates of the structure of N-terminal domain (2A8V^29^; left) and closed ring (2HT1; right) structures. The RNA oligonucleotide is shown in yellow and locations of G51V, G53V and Y80C are also shown as spheres with the same color code as in (a) and (b). (d) The secondary RNA binding domain is shown in the dimeric unit of the closed ring structure with the RNA (in yellow) bound at the interface of the two monomers (chains are shown in grey and black). P, Q and R loops are shown in green and the locations of Y274D, P279S/P279L, G324D and N340S are also indicated as spheres. Color codes for the mutations are same as in (a) and (b). (e) Distances of the positions of different mutations from important functional domains. The distances were calculated using the RASMOL program. For calculating the distances from primary (1°) and secondary (2°) RNA binding domains, the nearest RNA residues were considered and that from the P-loop, Cα atom of residue 184 was considered.

We mapped the positions of the mutations on the recently reported hexameric closed ring structure of Rho, which has both the primary and secondary RNA binding sites occupied with nucleic acids^19^ (Figure 2(a) and (b)). In general, the mutations are located within or close to the previously identified important functional domains of Rho. Among them, G51V, G53V and Y80C are in the primary RNA binding domain (Figure 2(c)). Y274D, P279S and P279L are in or close to the Q-loop (Figure 2(d)). G324D and N340S are close to the secondary RNA binding domain and G324D is in the R-loop (Figure 2(d)). The mutation I382N could not be located on the structure as the C-terminal end of the closed ring structure of Rho is not resolved. Interestingly, mutations in these important structural elements of Rho did not have a lethal phenotype.

It is revealed from the structure that amino acid Y80 makes direct contact with the nucleic acid in the primary RNA binding domain^29^ (Figure 2(c)). So there is a high probability that Y80C change will affect the primary RNA binding drastically. Amino acids G51 and G53 also come within 12–14 Å of the nucleic acid in the crystal structure and changes in these amino acids can also affect the primary RNA binding (see the distance calculations in Figure 2(e)). The crystal structure revealed the binding of only two nucleotides, whereas about ten nucleotides can be occupied in the primary RNA binding site of a monomer.^30^ Therefore it is likely that other amino acids of this domain (including G51 and G53) will take part in the primary RNA binding.^31^ Defect in the primary RNA binding due to the change in these three amino acids will subsequently affect the secondary RNA binding and the RNA release processes.

The amino acids G324 and N340 are situated close to the RNA in the secondary RNA binding site (Figure 2(d) and (e)). It is likely that G324, located in the R-loop, will take part directly in the interaction with RNA. Therefore, it can be predicted that changes in these two amino acids will cause a defect in the secondary RNA binding and in the ATP hydrolysis activities. This may in turn affect the processive translocation of Rho along the RNA, which will lead to a termination defect.

P279S and P279L changes in the Q-loop can alter the loop conformation by extending the length of the preceding helix. The closed ring structure of Rho revealed that the Q-loop is about 30 Å (Figure 2(e)) away from the nearest RNA residue in the secondary RNA binding site. In the closed ring structure of Rho, the Q-loop forms a hairpin-like structure from the disordered conformations observed in the open ring structure.^19^ This alteration may be important for attaining the active conformation of Rho and the change in the loop conformation due to the mutations will affect its function. On the other hand Y274, which is located just outside this loop, may come on the pathway of the RNA passing through the dimeric interface of two Rho protomers (Figure 2(d)). So Y274D may have similar defects as G324D and N340S changes.

In order to test these predictions of the additional defects of these mutants in different properties of Rho, we assayed their efficiencies for primary and secondary RNA binding as well as for their ATP binding and hydrolysis activities.

### Primary RNA-binding properties of the Rho mutants

There are two distinct types of polynucleotide interaction sites in Rho.^10,32^ The primary RNA-binding site, located in the N-terminal region, recognizes both single-stranded DNA and RNA, whereas the secondary binding site located at the central hole of the oligomer, binds specifically to the RNA. To investigate whether interactions in these two sites were affected by these mutations, we monitored the interactions specifically at the primary binding site by estimating the dissociation constant (*K*_d_) of each of the mutants for a 34-mer DNA oligonucleotide, (dC)_34_. Occupancy of a DNA oligomer only in the primary RNA binding site of the Rho crystal^15^ and protection of primary RNA binding domain by DNA oligomer in the protein footprinting studies,^10^ strongly suggest that DNA can specifically bind to this site and not to the secondary RNA binding site.

The dissociation constants (*K*_d_) for oligo(dC)_34_ of different Rho mutants were measured by both gel retardation and filter binding assays (Table 2, column 2). The *K*_d_ value for WT Rho was ∼ 10 nM. None of the mutants were defective for binding to oligo(dC)_34_ except Y80C, which did not show significant binding in both the gel retardation and filter binding assays. In order to check whether this defect is specific for a DNA oligo, we checked the efficiency of binding of a radiolabeled RNA oligo, rC_10_ to Y80C by UV cross-linking. This mutant showed significantly reduced cross-linking efficiency even at 20 μM of rC_10_ and did not show any gel-shift compared to WT Rho (data not shown). The binding defect of this mutation is consistent with the fact that this amino acid directly stacks against the base of the oligonucleotide^29^ (Figure 2(c)). Previously reported mutants in this region also exhibited similar defects in primary RNA binding.^33^

**Figure 3:**
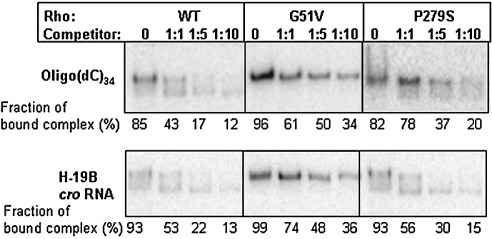
Stability of Rho–oligo(dC)_34_ complexes. Autoradiograms of native PAGE showing the amount of labeled oligo(dC)_34_ complexed with WT and different mutant Rho proteins survived in the presence of increasing concentrations of competitors: upper panel, unlabeled oligo(dC)_34_; lower panel, H-19B *cro* RNA. The fraction of bound complex was calculated as: [bound oligo]/[free oligo+bound oligo]. The 5 nM labeled oligo(dC)_34_ was used to form complex with 50 nM of WT and mutant Rho proteins.

G51V and P279S have about a threefold higher affinity for oligo(dC)_34_ compared to WT. Therefore the stability of the oligonucleotide-bound complexes of these two mutants in the presence of both single-stranded DNA and RNA as competitors was tested. Rho complexed with end-labeled oligo(dC)_34_ was challenged with increasing concentrations of either unlabeled oligo(dC)_34_ (Figure 3, upper panel) or H-19B *cro* RNA having a *rut* site (Figure 3, lower panel). It was observed that the G51V–oligo(dC)_34_ complex was significantly resistant to both the competitors compared to either WT or P279S complexes. This unusually stable interaction at the primary RNA binding site, which was not predicted from the crystal structure, could affect the proper release of RNA from the primary RNA binding site of G51V during the translocation process. It should be noted that the position of G51 is about 14 Å away from the nearest RNA residue (Figure 2(e)). Earlier it was reported that a termination defective Rho mutant G99V, in this same domain, also had a tighter (∼ 2.5-fold higher compared to WT Rho) primary RNA binding.^21^ Even though the amino acid P279 comes within 16 Å of the RNA in the primary RNA binding site (Figure 2(e)), it is difficult to envision from the available structural data why a mutation in Q-loop will increase the affinity for DNA in the primary RNA binding site. The distortion of the loop conformation due to this mutation might have an allosteric effect in the nearby primary RNA binding domain. In this context it is also to be mentioned that a high affinity primary RNA binding does not lead to a formation of a “super Rho”.

### Secondary RNA binding

Interaction of RNA in the secondary site is mandatory to activate ATP hydrolysis.^10,32^ It has been reported that due to the weak interactions at the secondary RNA binding site, it is difficult to observe the RNA binding at this site by using direct binding assays.^34,35^ So we measured the concentrations of poly(C), oligos rC_10_ (10-mer) and rC_25_ (25-mer) required to elicit half-maximal ATPase activities in the presence of excess ATP for estimating the binding efficiency to this site. For the oligos, rC_10_ and rC_25_, measurements were done in the presence of oligo(dC)_34_ at the primary site, because short RNA oligos (rC_5–8_) can elicit ATPase activity by binding to the secondary site only in the presence of oligo(dC).^14,36^

None of the mutants showed a significant defect in utilizing poly(C) as substrate (Table 2, column 3). Only P279S and G324D showed slightly less affinity compared to others. This was further supported by the fact that the rates of ATP hydrolysis with poly(C) were also not significantly different for the mutants (Table 2, column 4). It was observed that a strong substrate like poly(C) can mask the defects in secondary site binding,^12,37^ so we used two shorter RNA oligos, rC_10_ and rC_25_, to assess the binding defects in the secondary site. All the mutants, except I382N, failed to show significant amounts of ATP hydrolysis even at a very high concentration of the oligo, rC_10_ (Table 2, column 6). Even with the longer oligo rC_25_, the affinity for G51V was only increased, but still it showed ∼ 100-fold weaker affinity compared to WT (Table 2, column 5). In general, we concluded that except I382N, all the mutants were defective in secondary RNA binding or they had an extremely slow rate of ATP hydrolysis. This defect should have contributed significantly in their inability to terminate.

Most likely, the defect in binding of oligo (dC)_34_ to the primary site of the Y80C mutant did not stimulate the ATPase activity with the shorter oligos at the secondary site. In case of G51V, the unusual mode of binding of (dC)_34_ in the primary site, might have failed to elicit the allosteric effect in the secondary site. Earlier, the reported tight binding mutant, G99V, also had a similar defect in binding to oligo rC_10_.^21^ Defects of G324D and N340S for secondary RNA binding were predictable from their locations close to this site. Although according to the crystal structure^19^ (Figure 2(e)), amino acids Y274 and P279 are more than 20 Å away from the closest RNA residue in the secondary site, these data and the previously obtained protein footprinting results with Q-loop mutants^12^ strongly suggest that this region of Rho also takes part in interactions with the RNA in the secondary site.

### ATP binding

We measured the apparent dissociation constant (*K*_d,app_) of ATP for WT and mutant Rho proteins in the absence of the RNA cofactor by using the UV-cross-linking technique. The apparent affinity of ATP for mutants Y274D, P279S and G324D was observed to be reduced by five-to eightfold, whereas it was comparable to WT for mutants N340S, G51V and I382N (Table 3). Mutations in R and Q-loops might have allosteric effects on the proximal P-loop, the ATP binding site. Cross-talk between the R-loop and P-loop has also been proposed from the closed ring crystal structure of Rho.^19^ There was no significant cross-linking of ATP for the Y80C mutant.

Cross-linking efficiency of the radiolabeled ATP varied among different mutants to the extent that it was negligible for Y80C. Absence of cross-linking means that either the binding affinity for ATP is very poor or some unusual conformational changes due to the mutation has impaired the chemistry of cross-linking. As Y80C can hydrolyze ATP in the presence of poly(C) (Table 2) the mutant must have the ability to bind ATP. Therefore, we measured the *K*_m_ value of ATP for this mutant using poly(C) as cofactor and compared with the WT Rho (Table 3, data in parentheses). Y80C, indeed showed about fourfold increase in *K*_m_ value, which indicated that the binding of ATP has been affected due to this mutation. It is of interest to note that a mutation in the primary RNA binding domain can affect the conformations in the ATP binding domain which is located ∼ 40 Å away (Figure 2(e)).

### Y80C change in the N-terminal causes conformational changes in the distal C-terminal domain

Besides impairing the primary RNA binding, the Y80C mutation also caused significant defect in ATP binding. This led us to hypothesize that this change may cause conformational changes in the distal (∼ 40 Å) C-terminal domain allosterically. To test this hypothesis, we probed the conformations of WT and mutant Rho by limited proteolytic cleavages, fluorescence anisotropy and fluorescence quenching.

**Figure 4:**
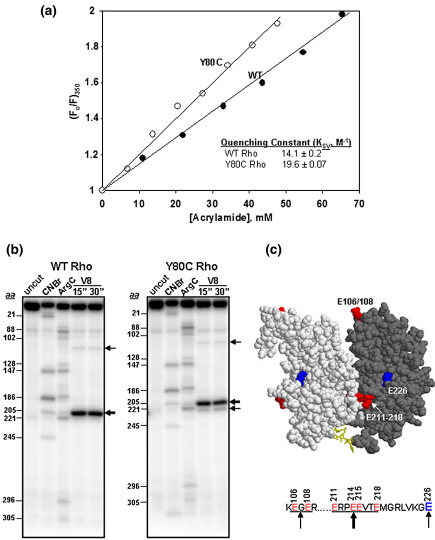
Conformational changes in the C-terminal domain of the Y80C mutant. (a) Stern–Volmer plots for acrylamide quenching of tryptophan fluorescence of WT and Y80C Rho proteins. The quenching constant (*K*_SV_) was calculated from these plots using the equation: (*F*_0_/*F*)_350_ = 1+ *K*_SV_[*Q*], where *Q* is the concentration of the quencher, acrylamide. (b) Limited proteolysis of end-labeled WT and Y80C Rho proteins with V8 protease. Glutamic acid residues corresponding to the major cleavage products are indicated by arrows. Thickness of the arrow corresponds to the intensity of the bands. These positions were identified from the calibration curve obtained from the molecular weight markers generated with CNBr and ArgC digestions of the same end-labeled Rho. The amino acid positions from N to C-terminal are indicated to the left of the gel pictures. The thick arrow corresponds to the sensitivity towards the glutamic acids residues between 211 and 218. Individual amino acids are not resolved. Position of 106/108 and 226 are also indicated by thin arrows. (c) Locations of the V8 sensitive residues are indicated on the dimeric unit of the closed ring structure of Rho. The red spheres are the V8 sensitive patch comprising of glutamic acid residues at 211, 214, 215 and 218. Also E106/108 is also indicated as red spheres in the N-terminal domain. E226, which specifically became sensitive in the Y80C Rho is shown as a blue sphere.

We monitored the surface accessibility of the single tryptophan residue (W381), located ∼ 15 Å away from the ATP-binding site, by the fluorescence quenching technique using acrylamide as a neutral quencher.^38^ This tryptophan residue emits a fluorescence signal at 350 nm upon excitation at 295 nm. Quenching of this signal was plotted against the increasing concentration of acrylamide to obtain the quenching constant (*K*_sv_) (Figure 4(a)). The value of *K*_sv_ increases as the tryptophan becomes more surface accessible. We observed that this tryptophan in the Y80C mutant is more surface accessible compared to the WT.

We used a fluorescent analogue of GTP, Tb-GTP, which is a complex of terbium chloride and GTP,^39,40^ to see the local conformational flexibilities at the ATP binding pocket. Here we assumed Tb-GTP will bind to the same ATP binding site as GTP is also a good substrate of Rho.^41,42^ We measured the anisotropy (*r*) of the Tb-GTP moiety upon binding to the ATP binding pocket. The anisotropy (*r*) of a fluorophore gives the measure of the rotational freedom of the species and reports the conformational flexibility of the surroundings.^39^ A higher value of *r* means less rotational freedom of the fluorescent probe. A lower value of *r* for Tb-GTP bound to Y80C compared to that obtained for WT Rho (Table 4) suggested more conformational disorders in the ATP-binding pocket.

In general, quenching constant and anisotropy values did not change drastically because of the Y80C mutation, which suggests that the conformational changes induced by the mutation in the C-terminal domain are more subtle. To further corroborate these data, we employed limited proteolytic digestions of the WT and Y80C Rho proteins to probe the conformational changes with V8 protease, which cleaves preferably at glutamic acid residues. We observed that a cluster of surface exposed glutamic acid residues (Figure 4(b)) near the dimeric interface of the C-terminal domain (Figure 4(c)) of both the WT and Y80C mutants were very sensitive to this protease. Interestingly, a new band corresponding to Glu226, close to this cluster (Figure 4(c) and (d)), was found to become sensitive to V8 digestion, specifically in the Y80C mutant. This observation further supports the proposal that the Y80C mutation in the primary RNA binding domain induces distinct but subtle conformational changes in the distal C-terminal domain, which might have affected the ATP binding domain and the surrounding regions.

### RNA-release efficiency of the Rho mutants from stalled elongation complex

**Figure 5:**
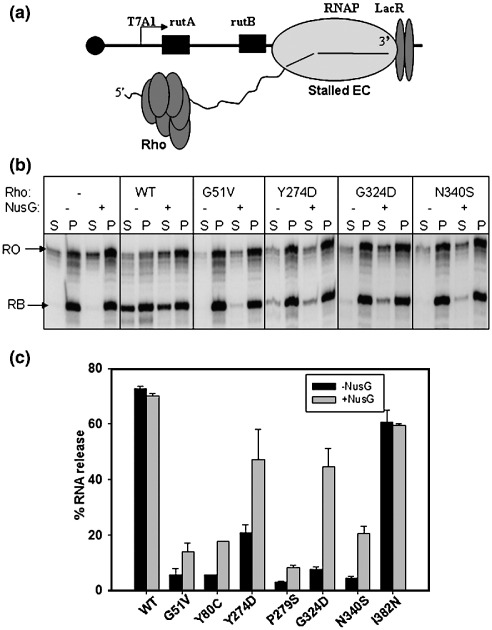
Rho-mediated RNA release from the stalled elongation complex. (a) Cartoon showing the design of a stalled elongation complex using lac repressor as a roadblock. In this set-up, the EC was stalled at 161 position of the *trp t'* terminator. Rho was added to the reaction after the stalled complex is formed and RNA release was measured from the supernatant. (b) Autoradiogram showing the amount of released RNA in the supernatant by WT and different Rho mutants both in the absence and presence of 200 nM NusG from the stalled EC. RB and RO denote the positions of roadblocked product and run-off product, respectively. The meaning of S and P is same as described for Figure 1. (c) The RNA release efficiencies shown by bar diagrams are calculated as: [2S]/[S+P].

**Figure 6:**
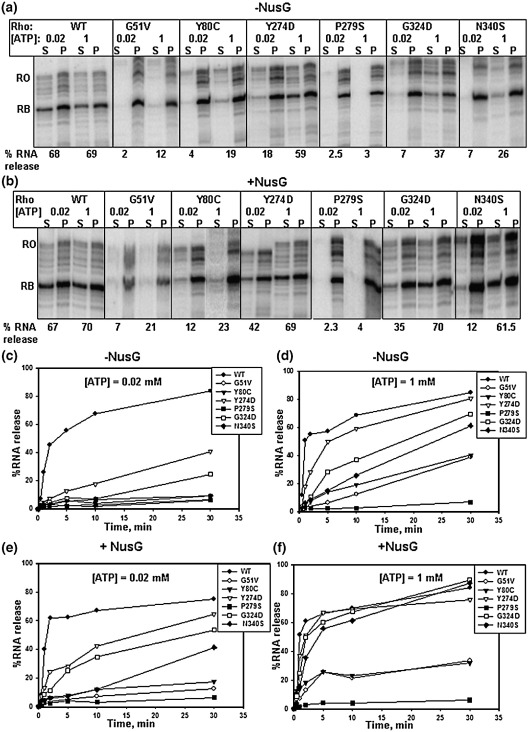
ATP and NusG dependence of Rho-mediated RNA release from a stalled elongation complex. Autoradiograms of the RNA release after 10 min of addition of different Rho mutants from the stalled EC (described in Figure 5) in the presence of different concentrations of ATP, both in the absence (a) and presence (b) of 200 nM NusG. (c)–(f) Curves showing the time courses of the RNA release upon addition of WT and different Rho mutants in the presence of indicated concentrations of ATP. The presence or absence of NusG in the reactions is also indicated. The RNA release efficiencies are calculated in the same way as described in Figure 5.

The majority of the Rho mutants are defective in secondary RNA binding, which might have affected the processivity during translocation along the nascent RNA and reduced the speed of translocation. We hypothesized that if their termination defect is due to slow translocation only, these mutants may be able to release RNA from the stalled ECs if sufficient time is allowed. So we stalled the EC on an immobilized template at a particular position within the *trp t'* terminator region (Figure 5(a)) using the lac repressor as a roadblock.^43^ The stalled elongation complex remains transcriptionally active and can restart transcription efficiently from this position upon removal of the lac repressor^43^ (data not shown). Under this condition the mutant Rho proteins will eventually be able to “catch up” the stalled EC if sufficient time is allowed and release the RNA. Also in the stalled EC set-up, one can monitor only the Rho-dependent RNA release uncoupled from the transcription elongation process and therefore the role of ATP and/or NusG on the different steps of the RNA release can be studied (see Figure 6).

To the stalled EC, WT or different mutant Rho proteins were added and the released RNA in the supernatant was monitored in the presence and absence of NusG. Under this experimental condition, WT Rho released ∼ 70 to 80% of the RNA from the stalled EC. Considering the non-specific rebinding of the released RNA to the beads, these values suggest an efficient release of RNA from the stalled EC by Rho. In the absence of NusG, all the mutants except I382N were still defective in releasing RNA even from a stalled EC. However, in the presence of NusG, a partial restoration in RNA release efficiency was observed for Y274D, G324D and N340S (Figure 5(b) and (c)), which is similar to that observed in continuous transcription assays (Figure 1(b), presence of NusG). This partial restoration of the RNA release efficiency from the stalled EC in the presence of NusG suggests that Y274D, G324D and N340S, unlike WT Rho, are very much dependent on NusG for interaction with the EC and possibly they are defective in the step(s) involving the release of RNA from the EC.

### High concentration of ATP and the presence of NusG restore the RNA release activity of the mutants Y274D, G324D and N340S

In order to further identify the kinetic step(s) defective for these Rho mutants we followed the time-course of RNA release from the stalled EC in the presence of different concentrations of ATP. We varied the concentrations of ATP to change the translocation rate. We preformed stalled EC, immobilized on magnetic beads, removed all the NTPs by extensive washing and WT or different mutant Rho proteins were added in the presence of 0.02 mM, 0.1 mM (data not shown) and 1 mM ATP. The time-course of RNA release in the supernatant was followed for 30 min, both in the absence (Figure 6(a), (c) and (d)) and presence of NusG (Figure 6(b), (e) and (f)). The rate (slope of the curve) and the efficiency (maximum amount of released RNA) of RNA release remained the same for WT Rho at all the concentrations of ATP tested. A modest increment of rate but not the efficiency of RNA release was observed in the presence of NusG. RNA release efficiency by the P279S mutant did not improve significantly, even at 1 mM ATP and in the presence of NusG. Under the same conditions, a modest improvement in the RNA release efficiency was observed for G51V and Y80C mutants, but it never reached to the level of WT Rho. For Y274D, G324D and N340S, the efficiency of RNA release improved significantly when 1 mM ATP was present and NusG was absent. For these mutants, both the efficiency and rate of RNA release was observed to regain the WT Rho level in the presence of both 1 mM ATP and NusG.

Higher concentrations of ATP might have increased the rate of ATP hydrolysis and as well as improved the processivity of Y274D, G324D and N340S Rho mutants, which in turn increased the overall rate of translocation. This has enhanced the chances of the Rho mutants to be in the vicinity of the stalled EC. These mutants may also have defects in the RNA release step, which most likely requires a direct interaction with the EC. The presence of NusG helped them to overcome this defect, which is reflected in the faster RNA release (Figure 6(f)).

## Discussion

Here we report the isolation and characterization of the termination defective mutants of Rho from a random mutagenesis screen and attempt to find the involvement and importance of different functions of Rho in releasing RNA from the EC in light of the recent crystal structure of Rho.^19^ Different properties of the mutants are summarized in Table 5. All the termination defective mutants (except I382N) were found to be located in the previously identified functional domains, such as in the primary RNA binding domain and in the secondary RNA binding domain including Q-loop and R-loops. The termination defect of the mutants G51V, Y80C and P279S could not be overcome under the most relaxed conditions that have been tested, suggesting that the primary RNA binding domain and Q-loop are the most crucial elements for RNA release activity. These mutants are also defective for most of the other functions of Rho. The termination defects of the mutants (Y274D, G324D and N340S), which are mainly defective for secondary RNA binding and most likely for the translocase activity, could be restored under relaxed *in vitro* conditions. The functional defects of most of the mutants correlate with their spatial localization in the crystal structure. We also show that mutations in the primary RNA binding domain (Y80C) can affect functions and induce conformational changes in the distal C-terminal domain, which is not predictable from the structure of Rho. We did not observe any severe *in vitro* defect in I382N, which is not consistent with its *in vivo* phenotype. Probably a modest *in vitro* defect can be amplified under more stringent *in vivo* conditions.

Importance of the polynucleotide interactions in the primary RNA binding domain of Rho in modulating other activities of Rho, apart from recognizing the *rut* site, had been envisioned earlier.^21,33^ Here we found that a specific mutation in position Y80, which directly interacts with the RNA at the primary site, intrinsically makes Rho defective for ATP binding (Table 3), induces conformational changes in the ATP-binding pocket (Figure 4(a) and Table 4) and in the surrounding C-terminal domain (Figure 4(b)). As the defect in ATP binding and other conformational changes occurred in the absence of RNA binding we propose that this mutation by itself has induced a major allosteric effect in the C-terminal domain, which is located far away from primary RNA binding domain. On the other hand, mutation at G51, located in the same primary RNA binding domain, renders very stable binding with the nucleic acids (Table 2 and Figure 3). This leads to severe defects in the RNA release and secondary RNA binding. This suggests that it is important that Rho leaves the *rut* site once it starts tracking the nascent RNA. Based on the properties of these two point mutations in the primary RNA binding domain we propose that this domain of Rho not only works as an “eye” to find the *rut* site but also exerts allosteric control over the downstream interactions upon binding to RNA. Therefore, mutations in this domain block the subsequent steps in the process of RNA release. We have recently found that Psu, an inhibitor of Rho, which inhibits Rho-dependent termination by slowing down the rate of ATP hydrolysis, does not interact with the Y80C mutant.^44^ This lack of interaction between the Psu and Y80C mutant might also arise due to conformational changes in the ATP binding/hydrolysis domain.

What is the role of Q-loop? The Q-loop is a structural element, which is located at the entry point of secondary RNA binding domain and protrudes into the central hole of the Rho hexamer. A comparison of the open and closed ring structures of Rho reveals that this loop undergoes major conformational changes upon ring closure^19^ and was found to be protected in the Rho–poly(C) complex from the hydroxyl radical cleavage.^10^ P279S and earlier reported mutants in this loop^12^ are defective in secondary RNA binding and in subsequent activities like ATP hydrolysis and translocation. Based on these results and the spatial location of this loop in the Rho hexamer (Figure 4(c)), we propose that this structural element works as a rudder that guides the RNA into the dimeric interface of the secondary RNA binding domain. This proposal is consistent with the “RNA handoff model”, where the Q-loop is proposed to be involved in transferring RNA between the subunits during translocation.^19^

It is known from the previous works that ATPase or NTPase activity of Rho is required for transcription termination^45,46^ and is believed that the free energy generated from the hydrolysis is required for the translocation of Rho along the RNA.^47,48^ Using a roadblocked EC we have uncoupled the transcription elongation from the Rho-dependent RNA release from the EC, which enabled us for the first time to observe a direct correlation between ATP hydrolysis and RNA release by Rho from a stalled EC. The RNA release efficiency and the rate of RNA release improved at high concentration of ATP for the three mutants (Y274D, G324D and N340S) in the secondary RNA binding domain (Figure 6). These results suggest that a higher rate of ATP hydrolysis improved their processivity and overall rate of translocation and the free energy from the ATP hydrolysis is used to bring them in the vicinity of the EC. It is likely that the ATP hydrolysis helps Rho to remain bound to the nascent RNA during the “chase”, because intrinsically the interactions in the secondary RNA binding domain are weak.

On the other hand, RNA release kinetics experiments (Figure 6) suggest that NusG is involved only in the RNA release step(s) once Rho reaches the vicinity of the EC, which is consistent with role of NusG that was proposed earlier.^49^ WT Rho on its own is capable of releasing RNA, but the speed of RNA release is increased in the presence of NusG. Early termination that is usually observed in the presence of NusG (Figure 1(a)) is the consequence of this enhanced rate of RNA release. So the requirement of NusG becomes mandatory under *in vivo* conditions where the RNA release has to be performed within a small time window. The RNA release step, in addition to their translocation defect, must be severely compromised in case of the mutants Y274D, G324D and N340S and therefore they are highly dependent on NusG. The final RNA release step may involve a direct interaction of Rho with the EC, so it is possible that the region of Rho defined by these amino acids is involved in the interaction with the EC.

Based on the properties of different termination defective mutants of Rho and in the light of open and closed ring hexameric structures complexed with nucleic acids in primary and secondary RNA binding domains we sum-up the sequential steps that lead to the RNA release from the EC. (1) The primary RNA binding domain binds RNA and forms an open ring hexameric Rho. This interaction also involves conformational changes in the C-terminal domain. (2) RNA gets channeled into the secondary RNA binding domain *via* the Q-loop and the closed ring structure is formed. (3) This activates ATP hydrolysis and onsets the translocation of Rho along the mRNA, which brings Rho in the vicinity of the EC. (4) Rho then releases RNA possibly by pushing the EC or by pulling the RNA out of the EC by its translocase activity. It is also possible that Rho specifically interacts in the RNA exit channel and exerts allosteric effects on the stability of the EC. As NusG is only involved in increasing the rate of RNA release at this step, it is possible that NusG in the presence of Rho makes the EC more prone to termination.

How important is Rho-dependent termination *in vivo*? All the termination defective mutants of Rho that we have isolated are viable. Among them, Y80C is defective for most of the functions of Rho. Surprisingly, these apparent severe defects did not cause lethality to the cells. This raises the question, that how important is the process of Rho-dependent termination when the cells are growing exponentially and why Rho is essential to *E. coli*. We speculate that due to the multi-functional nature of Rho, it may very well have other essential function(s), which is more important than transcription termination.

## Materials and Methods

### Biochemicals and enzymes

NTPs, poly(C), [γ-^32^P]ATP (6000 Ci/mmol, 3000 Ci/mmol and 30 Ci/mmol) and [α-^32^P]CTP (3000 Ci/mmol) were purchased from Amersham. Antibiotics, IPTG, lysozyme, DTT and BSA were from USB. Primers for PCR, oligo(dC)_34_, rC_10_, rC_25_, galactose, Protein kinase A, acrylamide and TbCl_3_ were obtained from Sigma. Restriction endonucleases, polynucleotide kinase and T4 DNA ligase were from New England Biolabs. WT *E. coli* RNA polymerase holoenzyme was purchased from Epicenter Biotechnologies. *Taq* DNA polymerase was from Roche. *Staphyloccocal aureus* V8 protease and Sub-maxillary protease (Arg-C) were from Pierce. CNBr was obtained from Fluka.

### Strain construction

Bacterial strains, plasmids and phages used in this study are listed in Table 6. *trpE9851*(Oc)^50^ was moved by P1 transduction from GJ3183 into GJ3192 and designated as RS336. The P_*lac*_-H-19B *nutR/T_R1_-lacZYA* cassette obtained from pK8628^22^ was inserted as single copy at the phage λ attachment site of strain RS336, resulting in strain RS364. Similarly, single copy fusion without H-19B T_R1_ rho-dependent terminator (P_*lac*_-*lacZAY* from pRS431) was constructed and designated as RS391. *rpoB8*^25^ and *rpoB2*^25^ genes were also moved into GJ3192 and named as RS446 and RS451, respectively. P_*lac*_-H-19B *nutR/T*_*R1*_-*lacZYA* and P_*lac*_-*lacZAY* were inserted into RS446 in single copy resulting in the strains RS449 and RS450, respectively. Strain RS445 was constructed by single copy insertion of P_*lac*_-*lacZAY* in GJ3161.

### Random mutagenesis and screening of Rho mutants

The plasmid pHYD567 with the WT *rho* gene was transformed into XL1-Red mutator strain (Stratagene). The mutagenized plasmid library was isolated and electroporated into the background strain RS336 with a chromosomal deletion in the *rho* gene. In this strain, Rho is supplied from a shelter plasmid carrying WT *rho* gene, which has an IPTG dependent conditional replicon (pHYD1201).

The transformants were plated on MacConkey agar plates supplemented with 1% (w/v) galactose and appropriate antibiotics. The strain was propagated in the absence of IPTG to remove the shelter plasmid so that the sole supply of Rho will be from the mutagenized plasmid library. The *galEP3* mutation in the strain confers Rho-dependent transcriptional polarity on the downstream genes, so that the cell is unable to utilize galactose to satisfy its auxotrophy in the presence of a functional Rho.^51^ Rho mutants can release polarity in such a strain and can make the strain able to utilize galactose. *gal*^+^ transformants were picked, purified three times by streaking in the same medium.

Mutant strains were streaked on a minimal anthranilate plate to confirm the mutations. The *trpE9851*(Oc) mutation confers Rho-dependent transcriptional polarity on the downstream *trpCDBA* genes, so that the cell is unable to utilize anthranilate in the presence of functional Rho. The strain with WT Rho is incapable of growing on a minimal anthranilate plate while Rho mutant strains can overcome the polarity of the *trpE9851*(Oc) mutation. The putative Rho mutant plasmids were isolated and re-transformed into the background strain for ensuring the mutant phenotypes. The full-length *rho* gene was sequenced from each of these plasmids to identify the mutation and to ensure that no other mutations were present in the gene.

### Measurement of *in vivo* termination

The strains RS364 and RS391 were transformed with the mutants and WT Rho plasmids to estimate the *in vivo* termination efficiency at the H-19B *T*_*R1*_ rho-dependent terminator. Similarly RS449 and RS450 were transformed with the mutants and WT Rho plasmids to get the activities with B8 RNAP. The strains GJ3073 and RS445 were used to get the β-galactosidase activities in the presence of WT Rho. The measurements of β-galactosidase activities were done in a microtiter plate using a Spectramax plus plate reader following the published procedure.^52^

### Cloning and purification of wild-type and mutants of Rho, NusG and B8 RNA polymerase

The WT and mutant *rho* genes were PCR amplified using proof-reading DNA polymerase from pHYD567 and its derivatives (Table 6) and were cloned into the XhoI/NdeI site of pET21b to introduce a His-tag at the C-terminal end. All the mutant (except I382N) plasmids were then transformed into the BL21(DE3) over expressing strain. I382N was over expressed in salt-inducible strain BL21SI (Invitrogen). After induction, His-tagged WT and mutant Rho proteins were purified using Ni-NTA beads (Qiagen) as per the manufacturer's protocol. The proteins were eluted in the presence of 500 mM immidazole and further purified by passing through a heparin-Sepharose column (Amersham). The expression levels for all the mutants, except Y80C, were high and the majority of the proteins were present in the soluble fraction. As the expression level of Y80C was low, to avoid the contamination from the WT Rho expressed from chromosome, the purification was done in the presence of 1 M NaCl. For all the mutants the contamination from WT Rho protomers was also checked by passing the mutant Rho proteins through Ni-NTA columns in the presence of 1 M NaCl. Due to the dissociation of the subunits at high salt, non-His tagged WT protomers, if present, would be eluted in the wash-fractions only. We estimated that the contamination from the WT protomer was less than 5% for all the mutants. In all experiments concentrations of Rho is expressed in terms of hexamer. Cloning and purification of NusG has been described.^53^ B8 RNAP was purified according to the published protocol.^54^

Solubility of mutant Rho proteins was reasonably good and we did not observe any aggregation over the period of performing the experiments. Chemical cross-linking showed that like WT Rho,^55^ all the mutant Rho proteins remained as hexamer above the concentration of 1 μM (data not shown). CD spectra of the different mutants were similar to that of the WT Rho, indicating that the overall structural integrity of the mutants was maintained during the course of the experiments (data not shown). The composition of the different secondary structural elements for WT and mutant Rho proteins are as follows. α–Helix, 24–27%; β–sheet, 24–32%; turn, 13–17%; random coil, 28–32%. So the differential activities and loss of specific functions of the mutants in various assays were not due to instability of the mutants or presence of inactive fractions in the preparations.

### Gel retardation and filter retention assays

RNA binding to the primary RNA-binding site of the WT and mutant Rho proteins was measured by gel-retardation and filter retention assays using an end-labeled 34-mer oligo(dC). 10 nM of labeled oligo was used for the binding assays in the transcription buffer (25 mM Tris–HCl (pH 8.0), 5 mM MgCl_2_, 50 mM KCl, 1 mM DTT and 0.1 mg/ml of BSA) with the increasing concentrations of the WT or the mutant Rho (0.1 nM to 300 nM). The reactions were performed at 37 °C for 5 min before loading onto a 5% (w/v) native acrylamide gel. Fraction of bound species was quantified by Image Quant software of the phosphor-imager Typhoon 9200. Gel-shift assays with labeled rC_10_ were also done in the same way. For the filter retention assays, 5 μl of reaction mixtures (as above) were spotted onto a nitrocellulose membrane in duplicate and kept in a dot-blot apparatus (Bio-Rad). Each spot was washed with 500 μl of transcription buffer under vacuum. The fraction of bound oligo(dC)_34_ was estimated from the ratio of the intensity of the washed (retained) and unwashed (total) spots using Image-Quant software. The dissociation constant values were calculated by hyperbolic fitting of the binding isotherms. For the competition assays, 10 nM oligo(dC)_34_ was allowed to bind to 50 nM of WT or mutant Rho proteins for 5 min at 37 °C followed by adding 10 nM (1:1), 50 nM (1:5) and 100 nM (1:10) of either cold oligo(dC)_34_ or H-19B *cro* RNA and incubating the reaction for further 5 min at 37 °C. The amount of bound fraction that survived the competitor was estimated from the band intensities.

### ATPase assays

ATPase activity of the WT and mutant Rho proteins was measured from the release of inorganic phosphate (Pi) from ATP after separating on the polyethyleneimine TLC sheets (Macherey-Nagel) using 0.75 M KH_2_PO_4_ (pH 3.5) as the mobile phase. In all the ATPase assays the composition of the reaction mixtures was 25 mM Tris–HCl (pH 8.0), 50 mM KCl and 5 mM MgCl_2_, 1 mM DTT and 0.1 mg/ml of BSA. The reactions were stopped with 1.5 M formic acid at the indicated time points. To determine the concentration of poly(C) required for half-maximal ATPase activity (Table 2, column 3), 25 nM Rho was incubated with different concentrations of poly(C) at 37 °C. Reaction was initiated with 500 μM ATP together with [γ-^32^P]ATP (6000 Ci/mmol) and was stopped after 15 min by formic acid. Release of Pi was analyzed by exposing the TLC sheets to a Phosphorimager screen for 5 min and subsequently by scanning using Typhoon 9200 (Amersham) and quantified by Image QuantTL software. The concentration of poly(C) corresponding to half-maximal ATPase activity was determined by fitting the plot of amount of Pi release against concentration of poly(C) to a sigmoidal curve. To determine the concentration of rC_10_ and rC_25_ corresponding to half-maximal ATPase activity (Table 3, columns 4 and 5), the titrations were done in the presence of 1 μM oligo(dC)_34_. The concentration of ATP was 1 mM. Other conditions were similar to those described above.

To calculate the *K*_m_ values of ATP for WT and Y80C mutant of Rho, in the same reaction mixture as described above, 5 nM of Rho was incubated with 10 μM of poly(C) at 30 °C for 10 min and ATP hydrolysis was initiated by the addition of different concentration of ATP (10 μM –100 μM). Aliquots were removed and mixed with 1.5 M formic acid at various time points. The product formation was linear in the time range (up to 5 min) that we used for calculating the initial rate of reaction. The initial rates of the reaction were determined by plotting the amount of ATP hydrolyzed *versus* time using linear regression method. Then the *K*_m_ values were determined from the double-reciprocal Lineweaver–Burk plots.

The rate of ATP hydrolysis in the presence of poly(C) was measured in the same reaction mixture as described above. The concentrations of Rho, ATP and poly(C) were 10 nM, 1 mM and 20 μM, respectively. Reactions were performed at 30 °C and aliquots were removed at different time points (at 30 s intervals up to 5 min) and mixed with 1.5 M formic acid.

### Photo-affinity labeling of ATP and rC_10_ to Rho

To determine the apparent dissociation constant (*K*_d,app_) of ATP, UV cross-linking of labeled ATP was performed with 50 nM Rho and varying concentrations of [γ-^32^P]ATP (30 Ci/mmol) in a 10 μl reaction mixture containing 25 mM Tris–HCl (pH 8.0), 50 mM KCl, 5 mM MgCl_2_and 1 mM DTT. The samples were irradiated for 5 min at room temperature in CL-1000 Ultraviolet cross-linker from UVP. This method of determining the apparent dissociation constant was used earlier.^14,34^ In a similar way the cross-linking of labeled rC_10_ to WT and Y80C mutant of Rho were performed. The samples were separated by SDS–PAGE, followed by scanning the gels in the phosphorimager Typhoon 9200 and quantified by Image-Quant software.

### Fluorescence quenching and anisotropy measurements

All the fluorescence experiments were performed in the buffer containing 10 mM Tris–HCl (pH 7.0) and 100 mM KCl at 25 °C in a Perkin Elmer LS 55 Luminescence spectrometer. Changes in the tryptophan (W381) fluorescence at 350 nm of WT and Y80C Rho were monitored upon exciting at 295 nm. Fluorescence quenching was measured in the presence of different concentrations of a neutral quencher, acrylamide. Normalized emission was plotted against increasing concentrations of acrylamide and the quenching constant (*K*_SV_) was obtained using Stern-Volmer equation: (*F*_0_/*F*)_350_ = 1+ *K*_SV_[*Q*], where, *F*_0_ is the initial fluorescence intensity and *Q* is the concentration of acrylamide.

TbGTP (3:1; 150 μM of terbium chloride and 50 μM of GTP) complex upon excitation at 295 nm gives rise to an emission signal characterized by two emission peaks at 488 nm and 545 nm.^39,40^ We measured the fluorescence anisotropies of this complex at 545 nm both in the absence and presence of either WT or Y80C Rho proteins.

### Limited proteolysis of Rho by V8 protease

The conformational changes induced by the Y80C mutation were probed from the limited proteolysis pattern generated by V8 protease. For the purpose of end-labeling the protein, we used heart muscle protein kinase (HMK) tagged WT and Y80C Rho. The tag was introduced at the C terminus by PCR amplification using appropriate primers and was cloned in pET21b vector. The resultant Rho proteins have both His and HMK-tags at the C-terminal end. They were purified in a similar way as described above and the termination assays showed that these two tags did not interfere with the function of the WT Rho. HMK-tagged Rho proteins were radiolabeled with [γ-^32^P]ATP (3000 Ci/mmol) using protein kinase A. The labeling reaction was done in buffer containing 20 mM Tris–HCl (pH 8.0), 150 mM NaCl, 10 mM MgCl_2_ and 10 μM ATP. 30 μg of Rho were incubated with 60 units of the kinase for 2 h at 21 °C. After labeling, 0.8 μM of labeled Rho were incubated with 0.05 μg of V8 protease in transcription buffer supplemented with 1 mM ATP for the indicated time at 37 °C. The reactions were stopped by adding 6X SDS-loading dye and were stored on ice. Samples were heated to 95 °C for 3 min, prior to loading onto a SDS–12.5%(w/v) PAGE. Gels were exposed overnight to a phosphorimager screen and were scanned using Phosphor-imager Typhoon 9200 and analyzed by Image-Quant software. Molecular weight markers of end-labeled Rho were generated by cyanogen bromide (CNBr) and Sub-maxillary protease (Arg-C) mediated cleavages. Methionine-specific cleavage reactions using CNBr contained (pH adjusted to pH2 with 1 M HCl) 0.8 μM of labeled Rho, 1 M CNBr and 0.4%(w/v) SDS in a 10 μl reaction. Reaction mixtures for arginine specific cleavage contained 0.8 μM of labeled Rho, 2 μg of Sub-maxillary protease and 0.1%(w/v) SDS in a 10 μl reaction. Reactions were terminated after 10 min at 37 °C by addition of 6X SDS-loading dye followed by boiling.

### Templates for *in vitro* transcription

Plasmid pRS106 containing *trp t'* terminator following a strong T7A1 promoter was constructed by replacing the H-19B *nutR*-triple terminator cassette from plasmid pRS25.^53^ Linear DNA templates for *in vitro* transcription were made by PCR amplification using the plasmid pRS106. In order to immobilize the template on streptavidin-coated magnetic beads, a biotinylated upstream primer was used. The lac operator sequence was incorporated in a downstream primer to make the templates with lac-operator at the downstream edge.^43^

### *In vitro* Rho-dependent transcription termination assay

*In vitro* Rho-dependent termination reactions were performed in T buffer (25 mM Tris–HCl (pH 8.0), 5 mM MgCl_2_, 50 mM KCl, 1 mM DTT and 0.1 mg/ml of BSA) at 37 °C. The template DNA was immobilized on the streptavidin-coated magnetic beads (Promega), wherever required, before starting the reaction. The reactions were initiated with 10 nM DNA, 40 nM WT RNA polymerase, 175 μM ApU, 5 μM each of GTP and ATP and 2.5 μM CTP to make a 23-mer elongation complex (EC_23_). [α-^32^P]CTP (3000 Ci/mmol) was added to the reaction to label the EC_23_. The complex was chased with 20 μM NTPs in the presence of 10 μg/ml of rifampicin for 5 min at 37 °C. 50 nM WT Rho or Rho mutants and 200 nM NusG were added to the chase solution as indicated. The reaction was stopped by adding 20 μl of phenol after 5 min of incubation at 37 °C and the released RNA was phenol extracted. RNA was fractionated in a 10% sequencing gel. For the reactions with B8 RNAP, the enzyme concentration was 100 nM and the EC was chased with 100 μM NTPs.

### RNA release from stalled elongation complex by WT and Rho mutants

*trp t'* template with the lac operator sequence at position 161 was used to study RNA release from stalled elongation complex (RB) by WT and Rho mutants. In order to make the stalled roadblocked complex on streptavidin beads, lac repressor was added before addition of the chasing solution. EC_23_ was made first and then was chased with 20 μM NTPs and 10 μg/ml of rifampicin for 2 min to make the RB. This was followed by addition of 50 nM of either WT or mutant Rho proteins in the presence or absence of 200 nM NusG. The reaction was incubated at 37 °C for 5 min and the supernatant was separated from streptavidin beads on a magnetic stand to measure the released RNA from the EC (Figure 5).

For the kinetics of RNA release from the RB, under different concentrations of ATP both in the presence or absence of NusG, RB was at first formed at position 161 of the *trp t'* template as described above, except that EC_23_ was chased with 50 μM NTPs. The RB was then washed extensively to remove the unincorporated NTPs and was re-suspended in T buffer supplemented with different concentrations of ATP (Figure 6). Following which 50 nM WT Rho or Rho mutants with or without 200 nM NusG were added to it. Half of the supernatant (S) was removed at various time points and added to the equal volume of RNA loading dye (Ambion). The rest of the reaction (half of the supernatant + pellet; P) was phenol extracted and mixed with the dye.

## Figures and Tables

**Table 1:** *In vivo* termination defects of the Rho mutants

Rho alleles	WT RNAP β-Galactosidase (arbitrary units)						B8 RNAP β-Galactosidase (arbitrary units)		
	Δ*rho: kan*^a^			*WT rho*^b^			Δ*rho: kan*^c^		
	+*T_R1_*	−*T_R1_*	%RT	+*T_R1_*	−*T_R1_*	%RT	+*T_R1_*	−*T_R1_*	%RT
WT	42 ± 2	973 ± 28	4.3	37 ± 2	839 ± 68	4.4	78.5 ± 10	564 ± 83	13.9
G51V	334 ± 20	690 ± 24	49.0	360 ± 17	860.5 ± 62	41.8	445 ± 32	782 ± 128	56.9
G53V	548.5 ± 54	678 ± 22	80.9	433 ± 11	1055 ± 45	41.0	458 ± 48	636 ± 96	72.1
Y80C	435 ± 12	687 ± 11	63.3	422 ± 23	1072 ± 46	39.3	645 ± 60	850 ± 180	75.8
Y274D	605.5 ± 45	729 ± 34	83.1	324 ± 14	1183 ± 44	27.4	435 ± 45.5	623 ± 115	69.8
P279S	362 ± 8	677 ± 25	53.4	308 ± 6	870 ± 10	35.4	345 ± 42	633 ± 122	54.5
P279L	686 ± 39	1116 ± 39	61.5	317 ± 22	852 ± 42	37.2	772 ± 36	1304 ± 84	59.2
G324D	636.5 ± 24	1074 ± 52	59.3	298 ± 13	839 ± 33	35.5	847 ± 116	1127 ± 18.5	75.1
N340S	732 ± 45.5	1102 ± 35	66.4	284 ± 15.5	973 ± 33	29.2	1272 ± 93	1553 ± 275	81.9
I382N	750 ± 54	1158 ± 24	64.8	290 ± 11	1057 ± 24	27.5	1050 ± 33	1247 ± 182	84.1

These above strains were transformed with the plasmids bearing different WT and *rho* mutants. The ratio of β–galactosidase values in the presence and absence of *T*_*R1*_ terminator gives the efficiency of terminator read-through (%RT). This Rho-dependent terminator is derived from the *nutR-cro* region of a lambdoid phage H-19B and found to behave similar to the *nutR/T*_*R1*_ of λ phage. The averages of five to six independent measurements are shown.

a Strains RS364 and RS391 with WT RNA polymerase and deletion of *rho* in the chromosome.

b Strains GJ3073 and RS445 with WT RNA polymerase and WT *rho* in the chromosome.

c Strains RS449 and RS450 with B8 RNA polymerase and deletion of *rho* in the chromosome.

**Table 2:** *In vitro* primary and secondary RNA binding properties of the Rho mutants

Rho mutants	*K*_d_^a^ (dC)_34_ (nM)	[poly(C)] at half-maximal ATPase activity^b^ (μM)	ATPase activity on poly(C) RNA^c^ (nmol of ATP/min per μg Rho)	[rC_25_] at half-maximal ATPase activity^d^ (μM)	[rC_10_] at half-maximal ATPase activity^e^ (μM)
WT	10 ± 0.9	0.59 ± 0.02	29.1 ± 3.5	0.13 ± 0.01	13.2 ± 2.6
G51V	3 ± 0.3	0.46 ± 0.04	15.5 ± 0.2	15.45 ± 1.75	> 200
Y80C	> 3000	0.14 ± 0.06	20.4 ± 0.2	> 500	> 200
Y274D	12 ± 1.4	0.43 ± 0.02	14.1 ± 1.0	> 500	> 200
P279S	3 ± 0.1	0.78 ± 0.03	33.1 ± 2.3	> 500	> 200
G324D	10 ± 2	0.72 ± 0.05	19.6 ± 4.0	> 500	> 200
N340S	11 ± 2	0.51 ± 0.09	23.6 ± 1.4	> 500	> 200
I382N	12 ± 3	0.30 ± 0.02	20.4 ± 0.05	0.39 ± 0.01	32.3 ± 1.0

a *K*_d_ values were average of those obtained from gel shift and filter binding assays using end-labeled (dC)_34_. Fractions of bound complexes were plotted against the concentration of Rho and the plot was fitted to a hyperbolic binding isotherm to determine the dissociation constant. Concentration of oligo(dC)_34_ is expressed in terms of DNA ends. For Y80C there was no binding up to 3 μM.

b Amount of radiolabeled inorganic phosphate release from [γ-^32^P]ATP was plotted against the increasing concentration of poly(C). Concentration of poly(C) corresponding to the half-maximal ATPase activity was determined by fitting the plot to a sigmoidal curve. The concentration range of poly(C) was 0 to 5 μM, in terms of the nucleotides. The concentration of ATP was 500 μM. Poly(C) will bind both to the primary and secondary RNA binding sites. However, the measurements are based on the activities solely in the secondary RNA binding site, so the values obtained by this method will reflect binding defects, if any, in the secondary site.

c The initial rate of ATP hydrolysis in the presence of poly(C) as cofactor was determined by fitting the plot of the amount of un-hydrolyzed ATP against time to the equation *y* = *y*_o_ exp(-λt), where λ is the rate constant. The time-points in the linear region (up to 5 min) were considered for the calculations. Concentrations of ATP and poly(C) were 1 mM and 20 μM, respectively.

d Except for WT, G51V and I382N, a significant amount of ATPase activity was not observed up to 500 μM rC_25_. Concentration of rC_25_ is expressed in terms of RNA ends. The concentration of ATP was 1 mM.

e Except for WT and I382N, a significant amount of ATPase activity was not observed up to 200 μM rC_10_. Concentration of rC_10_ is expressed in terms of RNA ends. The concentration of ATP was 1 mM.

**Table 3:** ATP binding of the Rho mutants

Rho mutants	*K*_d, app_ (μM)
WT	0.54 ± 0.06
	(11.7 ± 2.2)
G51V	1.35 ± 0.26
Y80C	No cross-linking
	(43.2 ± 0.8)
Y274D	3.15 ± 0.26
P279S	4.28 ± 0.54
G324D	2.45 ± 0.15
N340S	1.09 ± 0.12
I382N	0.60 ± 0.1

Apparent dissociation constant (*K*_d,app_) of ATP was determined by UV-cross-linking. The intensities of the cross-linked species were plotted against increasing concentrations of [γ-^32^P]ATP and *K*_d_ values were obtained from the hyperbolic fitting of the plots. For Y80C, no cross-linking was observed up to 30 μM [γ-^32^P]ATP. *K*_m_ values of ATP for WT and Y80C are shown in parenthesis. The concentration of WT and mutant Rho was 50 nM in hexamer.

**Table 4:** Fluorescence anisotropy (*r*) values of Tb-GTP

Species	Anisotropy (*r*)
Free Tb-GTP [150 μM: 50 μM]	0.096 ± .005
Tb-GTP+ 100 nM WT Rho	0.246 ± .007
Tb-GTP+ 150 nM Y80C Rho	0.183 ± .003

In all the experiments, Tb-GTP complexes were made by incubating 150 μM Tb with 50 μM GTP. The majority of the Tb-GTP species were in bound form in the presence of the indicated amount of hexameric Rho. Anisotropies were measured at 25 °C.

**Table 5:** Summary of different properties of the termination-defective Rho mutants

Mutants	1° RNA binding	2° RNA^a^ binding	ATP binding	Suppression of termination defect^b^
G51V	++	+/−	++	No
Y80C	−	−	+^c^	No
Y274D	+	−	+	Yes
P279S	++	−	+	No
G324D	+	−	+	Yes
N340S	+	−	++	Yes
I382N	+	+	+++	Not defective *in vitro*
WT	+	++	+++	Not defective

All the activities of the mutants are expressed with respect to that of WT Rho.

a Binding activities are for rC_10_ and rC_25_ templates. +/−indicates ∼ 100 fold reduced binding on rC_25_ template.

b For the mutants Y274D, G324D and N340S the RNA release efficiency from the stalled EC improves to the level of WT in the presence of 1 mM ATP and NusG.

c For ATP binding of Y80C the *K*_m_ value is considered.

**Table 6:** Bacterial strains, plasmids and phages used in this study

Strain/plasmid/phage	Description	Source or reference
A. *Strains*		
GJ3192	MC4100 *galEp3* Δ*rho*::Kan^R^ with pHYD1201, Amp^R^	56
GJ3183	MC4100 *galEp3 trpE9851(Oc) zci-506::Tn10 nusG*-G146D *rho*-R221C	56
RS336	GJ3192, *trpE9851(Oc)* Tet^R^	This study
RS364	RS336, λRS45 lysogen carrying P_*lac*_ – H-19B *nutR-T*_*R1*_*-lacZYA*	This study
RS391	RS336, λRS88 lysogen carrying P_*lac*_ –*lacZYA*	This study
GJ3073	MC4100 *galEp3,* λRS45 lysogen carrying P_*lac*_ – H-19B *nutR-T*_*R1*_*-lacZYA*	Dr J. Gowrishankar
GJ3161	MC4100 *galEp3*	56
RS445	GJ3161, λRS88 lysogen carrying P_*lac*_ –*lacZYA*	This study
RpoB8	MG1655, *rpoB8* Tet^R^ Rif^R^	25
RS446	GJ3192, *rpoB8* Tet^R^ Rif^R^	This study
RS449	RS 446, λRS45 lysogen carrying P_*lac*_ – H-19B *nutR-T*_*R1*_*-lacZYA*	This study
RS450	RS 446, λRS88 lysogen carrying P_*lac*_ –*lacZYA*	This study
RpoB2	MG1655, *rpoB2* Tet^R^ Rif^R^	25
RS451	GJ3192, *rpoB2* Tet^R^ Rif^R^	This study

B. *Plasmids*		
pHYD567	3.3 kb NsiI fragment carrying *rho*^+^ cloned from λ phage 556 of Kohara library into PstI site of pCL1920 (pSC101; Sp^R^, Sm^R^)	56
pHYD1201	3.3 kb HindIII-SalI fragment carrying *rho*^+^ sub-cloned from pHYD567 into HindIII-SalI sites of pAM34 (pMB1; IPTG dependent replicon, Amp^R^)	56
pRS342	pHYD567-*rho* G51V, Sp^R^, Sm^R^	This study
pRS387	pHYD567-*rho* G53V, Sp^R^, Sm^R^	This study
pRS350	pHYD567-*rho* Y80C, Sp^R^, Sm^R^	This study
pRS397	pHYD567-*rho* Y274D, Sp^R^, Sm^R^	This study
pRS341	pHYD567-*rho* P279S, Sp^R^, Sm^R^	This study
pRS347	pHYD567-*rho* P279L, Sp^R^, Sm^R^	This study
pRS344	pHYD567-*rho* G324D, Sp^R^, Sm^R^	This study
pRS346	pHYD567-*rho* N340S, Sp^R^, Sm^R^	This study
pRS399	pHYD567-*rho* I382N, Sp^R^, Sm^R^	This study
pK8628	pTL61T with P_*lac*_ – H-19B *nutR(T*_*R1*_*)-lacZAY* fusion, Amp^R^	22
pRS431	pTL61T with P_*lac*_ – *lacZAY* by deletion of H-19B *nutR-T*_*R1*_between HindIII and BamHI from PK8628, Amp^R^	This study
pRS106	pT7A1 cloned at EcoRI/HindIII sites upstream of *trpt* cloned at HindIII/BamHI sites of pK8641, Amp^R^	44
pRS96	WT *rho* cloned at NdeI/XhoI site of pET21b, His-tag at C-terminal, Amp^R^	This study
pRS378	*rho* G51V cloned at NdeI/XhoI site of pET21b, His-tag at C-terminal, Amp^R^	This study
pRS381	*rho* Y80C cloned at NdeI/XhoI site of pET21b, His-tag at C-terminal, Amp^R^	This study
pRS433	*rho* Y274D cloned at NdeI/XhoI site of pET21b, His-tag at C-terminal, Amp^R^	This study
pRS377	*rho* P279S cloned at NdeI/XhoI site of pET21b, His-tag at C-terminal, Amp^R^	This study
pRS379	*rho* G324D cloned at NdeI/XhoI site of pET21b, His-tag at C-terminal, Amp^R^	This study
pRS380	*rho* N340S cloned at NdeI/XhoI site of pET21b, His-tag at C-terminal, Amp^R^	This study
pRS432	*rho* I382N cloned at NdeI/XhoI site of pET21b, His-tag at C-terminal, Amp^R^	This study
pRS119	WT *nusG* cloned at NdeI/XhoI site of pET21b, His-tag at C-terminal, Amp^R^	53
pRS236	*lacI* cloned at NdeI/XhoI site of pET21b, Amp^R^	This study
pRS338	WT *rho* cloned at NdeI/XhoI site of pET21b, HMK tag and His-tag at C-terminal, Amp^R^	This study
pRS602	*rho* Y80C cloned at NdeI/XhoI site of pET21b, HMK tag and His-tag at C-terminal, Amp^R^	This study

C. *Phages*		
λRS45		Dr J. Gowrishankar
λRS88		Dr Robert Weisberg
